# Hydraulic Expansion Joint Contact State of Heat Exchanger Based on New Contact Area Measurement Method

**DOI:** 10.3390/ma16237448

**Published:** 2023-11-30

**Authors:** Wenze Zhang, Jianwei Liu, Jianping Ma, Yulin He, Sunbing Wu

**Affiliations:** School of Mechanical and Electrical Engineering, Guilin University of Electronic Technology, Guilin 541004, China; zwz15234186554@163.com (W.Z.); majianping1229@163.com (J.M.); hyl_2005@126.com (Y.H.); wusunbing10@163.com (S.W.)

**Keywords:** tube–fin heat exchanger, hydraulic expansion joint, contact area, contact gap, expansion pressure

## Abstract

The contact state of a seamless internal threaded copper tube and an aluminium foil fin not only affects the heat transfer efficiency of a tube–fin heat exchanger but also seriously affects its service life. In this study, hydraulic expansion technology was used to connect the copper tube with an internal thread with a 7 mm diameter to the fin of the heat exchanger. The influence of the expansion pressure and pressure holding time on the contact state was analysed through experiments and finite element simulation, and the variation law of the two on the contact state was obtained. The contact state was characterised by the contact gap and contact area. In order to obtain the specific contact area value, a new method of measuring the contact area was developed to reveal the variation in contact area between the copper tube and the fin after expansion. The results show that the contact gap decreases with an increase in expansion pressure, while the pressure holding time remains the same. The contact area increases with an increase in expansion pressure, and the rate of increase slows. When the expansion pressure is 18 MPa, the average contact gap is approximately 0.018 mm. When the expansion pressure reaches 16 MPa, the contact area ratio is 91.0%. When the expansion pressure increases to 18 MPa, the contact area ratio only increases by approximately 0.6%. Compared with the influence of the expansion pressure on the increase in contact area, the influence of the pressure holding time on the contact area is lower.

## 1. Introduction

As a compact heat exchanger, the tube–fin heat exchanger is currently the main form of heat exchanger and is composed mainly of heat exchange tubes and fins [1]. The quality of the connection between the heat exchange tube and the fin significantly affects the heat transfer efficiency. The heat exchange tube used is primarily a seamless internal threaded copper tube. The expansion joints of the copper tube and fin include mechanical, hydraulic, explosive, and rubber expansion [2]. Mechanical expansion technology is mature and inexpensive; therefore, it is employed by most enterprises. However, mechanical expansion causes the inner wall of the copper tube thread to be pulled and squeezed, destroying the inner thread structure and affecting the heat transfer efficiency. However, the pressure of hydraulic expansion is uniformly applied to the inner wall of the heat exchange tube, which does not damage the internal thread structure of the copper tube, and hydraulic expansion technology has the advantage of high forming accuracy [3].

After the expansion assembly of the heat exchange copper tube and fin, such contact states as the contact gap and contact area, which are manifestations of the assembly interface, seriously affect the heat transfer efficiency between the heat exchange tube and fin [4]. Therefore, the contact state of the assembly interface must be studied to explore the interface behaviour of the heat exchange copper tube and fin to obtain the ideal contact state. Research on the contact form of the assembly interface between the heat exchange tube and the fin has mainly focused on mechanical expansion. The research shows that there is a local contact gap between the heat exchange copper tube and the fin, which is a nonuniform contact state. In 1996, Critoph et al. [5] found that a gap of 0.01 mm can increase the total resistance by 10%. In 2002, Eisherbini et al. [6] proposed an experimental observation method for the contact gap and tested the contact gap between a copper tube and an aluminium fin under brazing and expansion, respectively. In 2003, Kim et al. [7] studied a mechanical expansion assembly of heat exchangers under vacuum conditions and analysed the relationship between the contact gap and expansion process. The results show that the contact gap is strongly related to the diameter of the expansion tube, flanging length of the fin, and shape of the fin hole. In 2006, Jeong et al. [8] mechanically expanded a Φ7 mm seamless internal thread copper tube with hydrophilic aluminium foil fins. The relationship between the contact gap and the expansion process was studied, and the influence of the mechanical expansion bead diameter on the contact gap was analysed. In 2006, Cheng et al. [9] studied the relationship between the contact gap and the expansion mould and analysed the influence law of three mould structures (spiral, forward, and backward) on the contact gap. In 2010 and 2011, Tang et al. [10,11] used finite element and test methods to study the contact state of the mechanical expansion of tube–fin heat exchangers. The influence of the expansion rate of the heat exchange tube on the contact gap was analysed. The structure and processing technology of the fins were optimised to reduce the contact gap and improve heat transfer efficiency. In 2014, Dawid et al. [12] proposed an improved method of estimating the average value of the contact thermal resistance of finned tube heat exchangers and studied the temperature distribution of the finned tube contact based on computational fluid dynamics (CFD) simulations and experimental measurements.

In 2016, Shobhana et al. [13] used a liquid–gas finned tube heat exchanger as the research object and studied the contact heat transfer between fins and tubes to different degrees using numerical analysis. They found that the contact area between the fins and tubes had a significant impact on the overall performance. In 2020, Sahel et al. [14] studied the contact between heat exchange tubes of different shapes and fins and analysed the influence of heat exchange tubes of different shapes on heat transfer efficiency and fluid flow. Numerical research revealed that the heat transfer coefficient of circular tubes was approximately 18% higher than that of flat tubes, and the pressure drop generated under the same conditions was moderate (approximately 10%). In 2021, Marcin et al. [15] studied the influence of air gaps on heat transfer under periodic flow conditions through numerical simulations of symmetrical section plate fin tube heat exchangers. Blocking the heat flow by the gap reduces the heat transfer rate. The fin discontinuity in front of the tube causes the smallest reduction in the heat transfer rate in comparison with the ideal tube–fin contact, especially for thin slits. In 2021, Khizhov et al. [16] carried out thermal, hydraulic, and pneumatic tests on the tube bundle of the heat exchanger and presented full-size tube samples with different fin ratios. The formula for calculating the effect of fin ratio on heat exchanger efficiency was given. In 2022, Ravikumar et al. [17] improved the heat transfer rate by changing the existing fin shape to contact the heat exchange tube and increased the contact by replacing the flat fin with a splined fin (a fin for adding an annular heat sink to a cylindrical thin-walled tube). The results showed that the heat exchange efficiency of the splined fin increased by more than 5% compared with that of the flat fin. In 2022, Svaiful et al. [18] took tube fin heat exchangers as their research object. The effect of enhancing the ratio of convex strip surface to heat transfer and friction characteristic on the heat exchanger was studied. The study found that the enhancement of four convex strips around the tube on the fin surface can generate the value of the field synergy angle on fluid flow. In 2023, Zhang et al. [19] studied the application of machine learning based on neural networks and genetic algorithms in multi-objective optimisation of heat exchangers. Taking the tube fin heat exchanger as the research object, the air inlet velocity and the ellipticity of the tube were used as optimisation variables to obtain the optimal heat exchange performance and pressure drop performance. In 2023, Aigul et al. [20] reported the results of an experimental study on the influence of various types of fins on heat transfer processes and hydraulic resistance. Their results show that fins in the form of crosses and triangles are the most efficient in terms of heat transfer. However, they create the greatest hydraulic resistance. In 2023, Feng et al. [21] analysed the mechanical expanded joint process of a plate fin-and-tube heat exchanger and the geometry of the fin tube hole needed to reduce the thermal contact resistance between the fin and the base tube and improve the heat transfer capability of the tube fin. The post-expansion contact stress between the fin and the base tube was calculated using finite element simulation, and a correlation between the contact stress and the thermal contact resistance was established.

The above research mainly focused on the contact between the heat exchange tube and fin during mechanical expansion, and the contact was increased to improve the heat exchange efficiency by changing the shape of the heat exchange tube and fin. However, hydraulic expansion technology was used to connect the heat exchange tube and fin, and their contact area was measured experimentally. Research on the change in the contact area of the hydraulic expansion parameters has been relatively scarce. To study the contact state between the copper tube and the fin after hydraulic expansion, an effective and reliable contact area measurement method must be developed. It is important to obtain the influence law of the hydraulic expansion parameters on the contact state by observing the distributions of the contact gap and contact area.

In this study, the contact state of a hydraulic expansion joint between a copper tube with an internal thread with a 7 mm diameter and an aluminium foil fin was studied. The contact state between copper tube and fin under different expansion pressures and holding times was studied through experiments and finite element simulation. A new method of measuring the contact area was proposed, and contact gap and contact area measurement tests were carried out to obtain the specific quantitative values of the contact gap and contact area, which provided effective data support for the further study of the heat exchange efficiency under the hydraulic expansion technology of the tube fin heat exchange tube. The change rule of the contact state of the hydraulic expansion joint between the copper pipe and fin was studied looking at two aspects: contact gap and contact area. The minimum contact gap and maximum contact area of the copper tube and fin after hydraulic expansion were obtained considering the rule of contact state, and the best hydraulic expansion parameters were obtained according to contact state. The research results can provide a reference for the application of hydraulic expansion technology in the connection process of a tube fin heat exchanger.

## 2. Experimental Research

**Figure 1:**
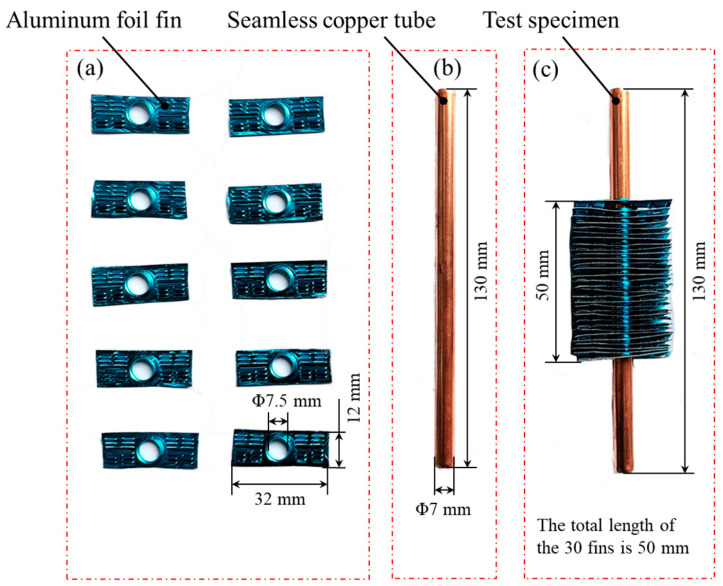
Experiment materials: (**a**) seamless internal threaded copper tube (TP2), (**b**) aluminium foil fins, and (**c**) experimental specimen.

The experiment of the contact state between a seamless internal thread copper tube and aluminium foil fin mainly consisted of two parts: the expansion experiment and the measurement experiment. The expansion test is the premise of a successful measurement experiment. The expansion experiment mainly involved the expansion of a copper tube and aluminium foil fin under different expansion pressure and pressure holding time parameters, and the sample required for the measurement experiment was made. The size of the contact gap and contact area can effectively reflect the contact state of the hydraulic expansion joint, so the measurement experiment was mainly divided into a contact interface gap observation experiment and contact interface area observation experiment. A seamless internal thread copper tube (TP2) and aluminium foil fins were used as test materials. The sample used in the experiment was composed of 30 single-hole aluminium foil fins and multiple pieces superimposed on a seamless internal thread copper tube (TP2) with a length of 130 mm and a diameter of 7 mm, as shown in Figure 1. The material characteristics of a seamless internal threaded copper tube (TP2) and hydrophilic aluminium foil fins are shown in Table 1 [22].

### 2.1. Expansion Experiment

**Figure 2:**
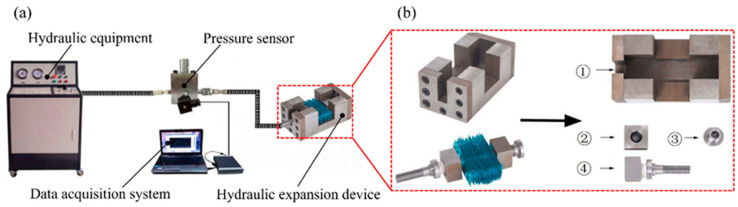
Expansion experiment platform: (**a**) heat exchanger hydraulic expansion system and device (in red box) and (**b**) heat exchanger hydraulic expansion device components: (1) expansion base, (2) threaded guide sleeve, (3) nut, and (4) liquid-filling hole stud.

An expansion experiment was conducted on the constructed experimental platform, and the hydraulic expansion device of the heat exchanger was independently designed, as shown in Figure 2a. The platform for the expansion experiment had three main parts: a hydraulic supply system, a data acquisition system, and a heat exchanger hydraulic expansion device. The main function of the hydraulic supply system was to provide the initial liquid pressure for the expansion of the copper tube and fin and to measure the change in the liquid pressure in the tube in real time through a data acquisition system. The structure of the independently designed hydraulic expansion device for the heat exchanger is shown in Figure 2b. The hydraulic expansion device of the heat exchanger was composed primarily of an expansion base, threaded guide sleeve, nut, and stud (with a liquid-filling hole). An O-shaped sealing ring was installed at the connection between the nut, stud, and copper tube.

The finite element simulation and heat exchanger expansion experiment were conducted using the dichotomy and trial-and-error methods. Reasonable parameters for the expansion pressure and pressure holding time were selected quickly, which laid the foundation for the next step in the contact state measurement experiment. During the expansion experiment, the copper tube broke when the expansion pressure reached 19 MPa. In the finite element simulation, the contact state did not change when the holding time reached 20 s. Therefore, the hydraulic expansion pressures were 12, 14, 16, and 18 MPa, and the pressure holding times were 0, 5, 10, 15, and 20 s. Combinations of 20 hydraulic parameters were used in the experiment. Three sample groups were prepared for each combination to avoid accidental experiments.

### 2.2. Measurement Experiment

#### 2.2.1. Contact Gap Observation Test

**Figure 3:**
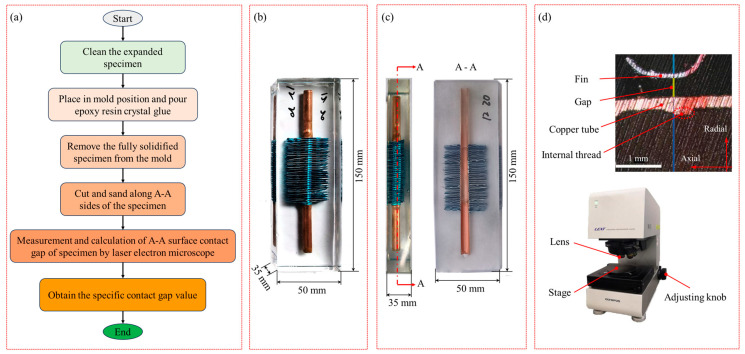
Contact gap test diagram: (**a**) specific experimental procedure, (**b**) sample after crystal glue pouring, (**c**) position of the A–A cutting face and specimen cut along the A–A face, and (**d**) expansion contact state diagram.

In order to better analyse the contact interface between the inner threaded copper tube and the aluminium foil fin after expansion, the contact interface was obtained after cutting the sample for observation. The specific test process is shown in Figure 3a. First, the sample connected between the expanded copper tube and the fin was cleaned, and the cleaned sample was placed in the pouring mould. Then, the prepared epoxy resin crystal drop glue was poured into the mould, so that the crystal drop glue completely covered the sample, and the sample was taken out after it solidified. The length of the poured sample was 150 mm, the width was 50 mm, and the height was 35 mm, as shown in Figure 3b. The pouring of the expanded specimen effectively solved the problem in which fins change the contact state and even fall off during the cutting process of the expanded specimen. Secondly, a CNC cutting machine was used to cut the solidified sample along the central axis of the A-A plane and polish the A-A cutting plane, as shown in Figure 3c. Then, a laser electron microscope with a high resolution up to 1 nm was used to take pictures of the central position of the cut sample for image acquisition and processing. The fin, the copper tube, and the gap between the copper tube and the fin can be clearly seen, as shown in Figure 3d. Finally, feature points were taken on the copper tube and fin to measure the gap distance of feature points. Equation (1) was used to calculate the average contact gap distance, where *L* is the average contact gap distance and *l_i_* is the distance between the copper tube and the characteristic point of the fin.

(1) $$L=\frac{\sum_{i=1}^{n}l_{i}}{n}$$

**Figure 4:**
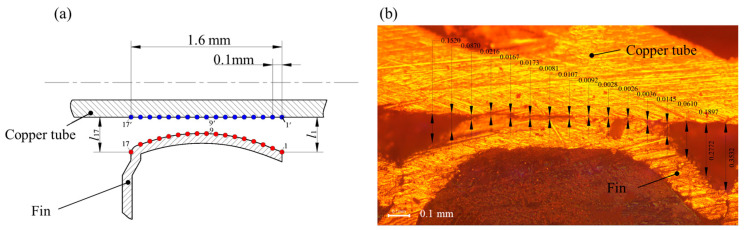
Contact gap measurement method: (**a**) position of copper tube and fin feature points (the red points are the feature points on the fin and the blue points are the feature points on the copper tube) and (**b**) contact gap measurement of interface image.

Regarding the selection of the position of feature points, when the distance between feature points was large, the calculated gap distance was relatively large, which was not conducive to showing the gap contrast difference. When the distance between feature points was relatively small, there were more measurement data and the processing data were relatively tedious to handle. Moreover, when the distance between feature points reached a certain distance, the large average contact gap was basically unchanged. It was found that when a feature point was taken on the copper tube and fin every 0.1 mm and the distance between the feature points was reduced, the average contact gap basically remained unchanged. The distribution position of feature points is shown in Figure 4a. There were 17 feature points in total. The gap distance measurement of the enlarged copper tube and fin interface image is shown in Figure 4b.

#### 2.2.2. Contact Area Observation Experiment

**Figure 5:**
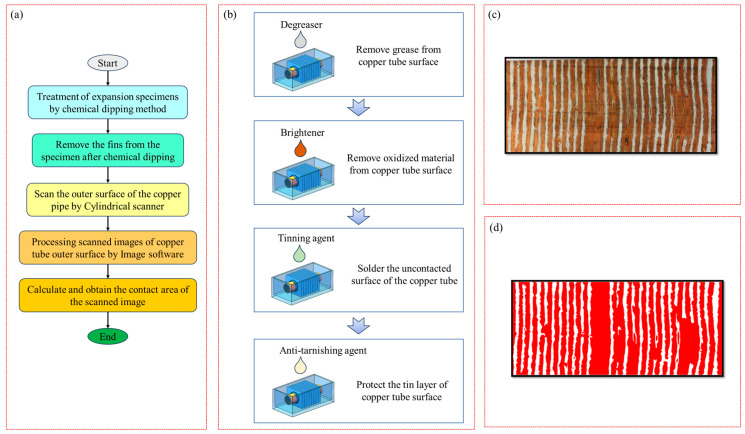
Contact area test diagram: (**a**) specific experimental procedure, (**b**) chemical dipping test procedure, (**c**) distribution of the contact site after chemical dipping and plating, and (**d**) distribution of the contact site after image processing.

After the copper tube with the internal thread and the aluminium foil fin expand, the aluminium foil fin and the copper tube with the internal thread may not be in full contact. Moreover, the flanging part of the fin covers the copper tube, and the contact area is relatively small. Therefore, it is difficult to measure the contact area between the two directly. Therefore, a chemical dipping method was developed in this study to measure the contact area between the internal threaded copper tube and the aluminium foil fin. The specific test process is shown in Figure 5a.

First, the expanded sample was chemically dipped and plated. The specific process is shown in Figure 5b: First, the expanded sample was put into the normal-temperature degreasing agent to degrease and remove oil from the surface of the internal thread copper pipe. For the second step, after oil removal, we put the sample into the copper-cleaning agent to clean it and remove the oxidising substances on the surface of the copper pipe so that the surface of the copper pipe was bright. In the third step, the cleaned sample was soaked in a copper-plating agent for 2 min to quickly obtain a bright silver-white tin coating on the surface of the non-contact internally threaded copper pipe. In the fourth step, the tinned sample was placed into an anti-discoloration agent and soaked for 2 min to protect the tinned surface and maintain a long silver-white appearance. Secondly, the fins on the surface of the sample were removed after chemical dipping. Then, the surface image of the copper tube was expanded and shot. The untouched tinned part of the copper tube was silver-white, and the untouched tinned part was brass, as shown in Figure 5c. Finally, ImageJ 1.53 software was used to process the expanded image of the copper tube, obtain the distribution of the contact parts between the copper tube and the fins, and calculate the contact area, as shown in Figure 5d.

## 3. Finite Element Analysis

**Figure 6:**
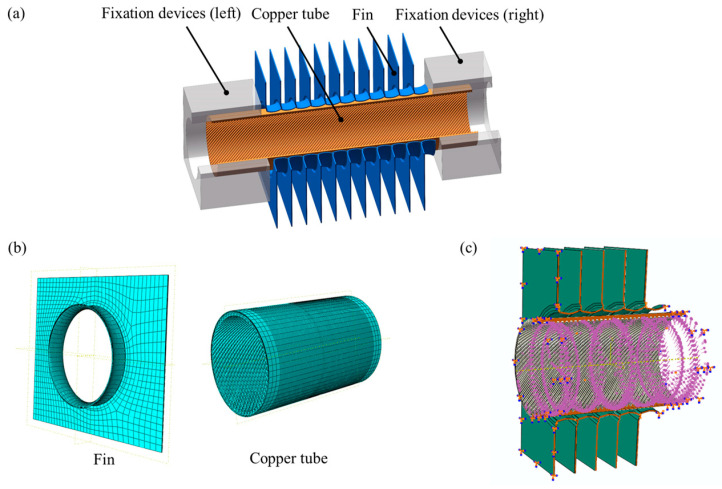
Finite element model: (**a**) geometric model, (**b**) mesh division, and (**c**) boundary condition setting.

The contact gap and contact area between the copper tube and the fin after expansion were obtained through the expansion and measurement tests. However, it was difficult to observe the change law of the contact between the copper tube and the fin during the expansion process, and it was difficult to control the accuracy of the pressure holding time during the test; therefore, ABAQUS 6.14 software was used for finite element simulation. The geometric model of finite element simulation is shown in Figure 6a. The geometric dimensions and materials of the internally threaded copper pipe and aluminium foil fins in the finite element model were consistent with the above tests.

### 3.1. Grid and Boundary Condition Settings

Mesh refinement was carried out on the position where the copper tube and fin came into contact, and the mesh size of the mesh refinement part was 0.1 mm, as shown in Figure 6b. There were 10,350 mesh units for each fin and 42,222 mesh units for each copper tube, and the cell type was C3D8R. The copper tube and fin were positioned using the positioning ring, with the positioning ring set to full restraint, and the copper tube and fin were set to free restraint, as shown in Figure 6c (the positioning ring is hidden in Figure 6c). The positioning ring was set as a rigid unit to reduce grid division. The copper tube and fin were set as elastoplastic deformable. Two positioning rings were used to fix the two ends of the pipe, and the dynamic friction coefficient between the copper pipe and the fin was set to 0.12.

### 3.2. Load Application and Analysis Step Setting

The radial liquid pressure load was applied on the inner wall of the copper pipe by setting the pressure to evenly distributed. Three analysis steps were designed. The first analysis step had a time of 0–1 s, that is, the pressurisation stage when the expansion pressure increased from 0 MPa to the maximum. The second analysis step time was 1–6 s, that is, the pressure holding stage in the expansion process of the copper tube and fin. The third analysis time was 6–8 s, that is, the unloading stage when the expansion pressure was unloaded from the maximum pressure to 0 MPa. The loading curve was consistent with the above test process. In addition, the equivalent stress–strain relationship, represented by Hollomon’s equation, *σ = Kε^n^*, was used for the finite element analysis. The relevant mechanical parameters of the copper pipe and fin input in ABAQUS 6.14 software are shown in Table 1.

## 4. Results and Discussion

### 4.1. Variation in Contact Gap

**Figure 7:**
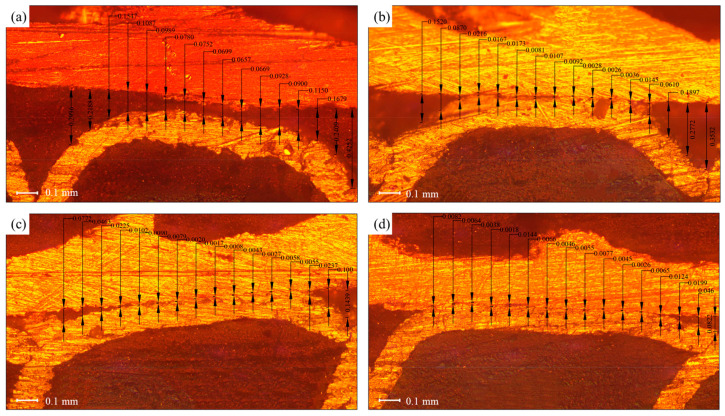
Clearance distance between copper tube and fin when the pressure holding time was 20 s: expansion pressures of (**a**) 12, (**b**) 14, (**c**) 16, and (**d**) 18 MPa.

Figure 7 shows a measurement diagram of the contact gap between the copper tube and the fin under different expansion pressures at a holding time of 20 s. As shown in Figure 7, when the expansion pressure was 12 MPa, there was no contact between the copper tube and the fin. The minimum clearance distance was approximately 0.0657 mm at the middle of the fin flanging, the maximum clearance distance was approximately 0.425 mm at feature point 1 at the front end of the fin flanging, and the clearance distance at feature point 16 at the back end of the fin flanging was approximately 0.299 mm. The gap experiment revealed that when the gap distance was less than 0.01 mm, the copper tube and the fin came into contact [5]. With an increase in the expansion pressure, the middle part of the fin flanging first made contact and then extended to both ends. When the expansion pressure reached 18 MPa, the maximum clearance distance occurred at feature point 1 of the front section of the fin flanging, which was approximately 0.082 mm, and the basic contact was at feature point 16 of the back end of the fin flanging.

**Figure 8:**
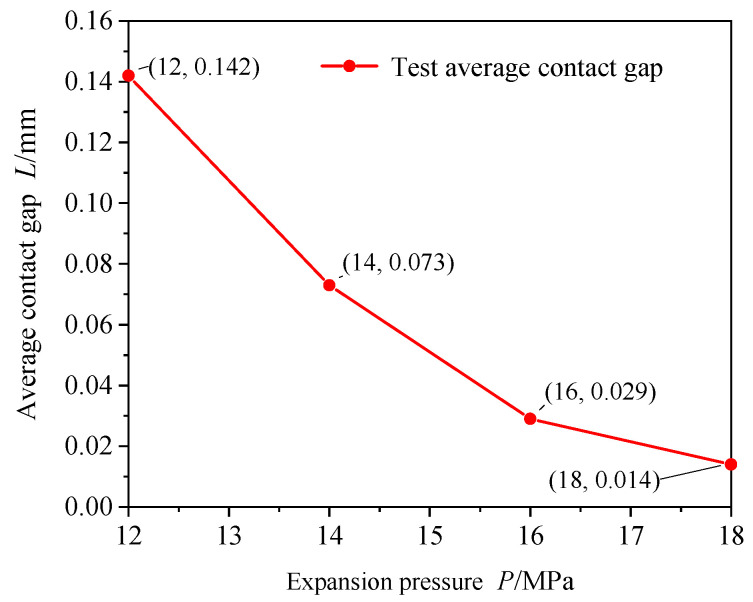
Influence law of expansion pressure on average contact gap (pressure holding time of 20 s).

The average gap distance was calculated using the gap distance of each feature point shown in Figure 7, and the relationship between the expansion pressure and the average gap distance was plotted, as shown in Figure 8. Under the condition of a constant pressure holding time, the average contact gap decreased with an increase in the expansion pressure, and the decreasing rate gradually lessened. When the expansion pressure was 12 MPa, the average contact gap was 0.142 mm. When the expansion pressure was 18 MPa, the average contact gap was 0.014 mm. When the expansion pressure increased from 12 to 18 MPa, the average contact clearance decreased by approximately 90%. When the expansion pressure increased by 2 MPa, the average contact gap decreased by approximately 48–60% of the original gap.

**Figure 9:**
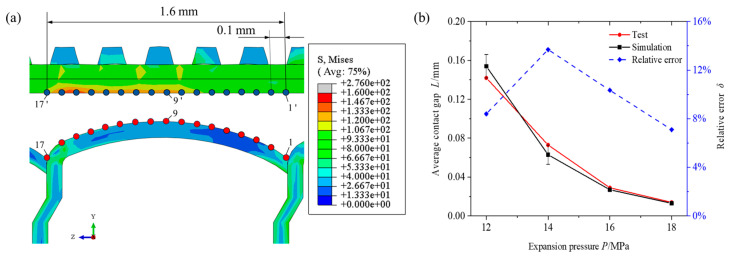
Verification of simulation model: (**a**) distribution of characteristic points in finite element simulation (the red points are the feature points on the fin and the blue points are the feature points on the copper tube) and (**b**) comparison of average contact gap between test and simulation.

Owing to the large error in the accuracy of the experimental method for controlling the pressure holding time, it was difficult to realise a period in which the pressure holding time was 0–5 s. Therefore, the change law of the expansion pressure and pressure holding time on the contact gap between the copper tube and the fin was studied using finite element simulation. To verify the accuracy of the finite element model, the geometric model size of the finite element was designed to be the same as that used in the experiment, and the forces of the copper tube and fin were the same. To ensure the accuracy of the comparison between the finite element simulation and the test, the same location and number of feature points were selected in the finite element simulation using the same method as that used in the test. The distances between feature points were averaged as the contact gap between the copper tube and the fin, as shown in Figure 9a. The gap distance curves between the copper tube and the fin for the finite element simulation and test are shown in Figure 9b. The relative errors of the test data of the contact gap and the finite element analysis results are shown in Table 2.

The average contact gap calculated using the finite element method was slightly smaller than the test result. The relative error between the experimental and finite element analysis results was less than 15%, and the gap size distribution between the fin and the copper tube was basically the same. Therefore, the finite element model used in this study exhibited high accuracy.

**Figure 10:**
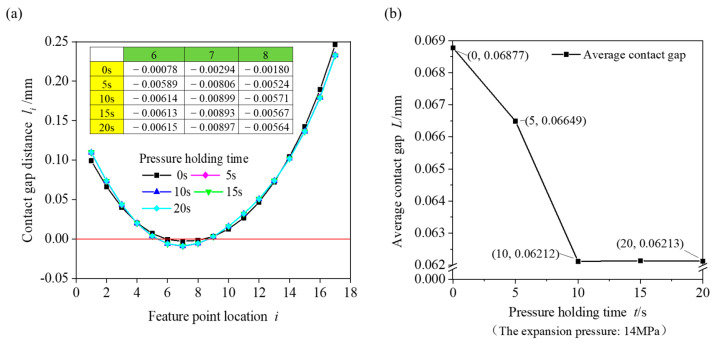
Contact gap changes simulated using the finite element method: (**a**) gap distance of feature points (the table shows the variation in the gap distance of feature points (in green) 6, 7 and 8 with the pressure holding time (in yellow)) and (**b**) average contact gap of feature points (expansion pressure: 14 MPa).

The contact gap distance between the heat exchange tube and fin with the representative expansion parameter combination (expansion pressure of 14 MPa, holding pressure time of 0–20 s) was analysed, and the gap change is shown in Figure 10. As shown in Figure 10a, the contact gap between feature points decreased when the pressure holding time increased from 0 s to 20 s. The table in Figure 10a shows the change value of the gap distance between the three feature points under the pressure holding time. The distance between the contact gaps at the positions of feature points 6–8 was negative, and the contact gap cannot be negative. The negative value here indicates that contact interference occurred between the copper tube and the fin in the simulation. When the holding time was 0 s, the contact gap of feature point 7 was −0.00294 mm. When the pressure holding time was 10 s, the contact gap of feature point 7 was −0.00899 mm. As the pressure holding time increased, the contact interference gradually increased, and the copper tube and fin maintained close contact. When the pressure holding time was 10–20 s, the contact gap at feature point 7 did not change and remained stable. Figure 10b shows the relationship between the pressure holding time and the average contact gap when the expansion pressure was 14 MPa. When the holding time was 0 s, the average contact gap was 0.06877 mm. When the pressure holding time was 10 s, the average contact gap was 0.06212 mm, which was a decrease of 9.67%. When the pressure holding time was 10–20 s, the average contact gap changed only slightly. The average contact gap could be reduced by maintaining the pressure between the expanded copper tube and the fin. The average contact gap was relatively large when the pressure holding time was 0 s, and the influence of the holding time on the average contact gap was relatively small when the holding time exceeded 10 s.

**Figure 11:**
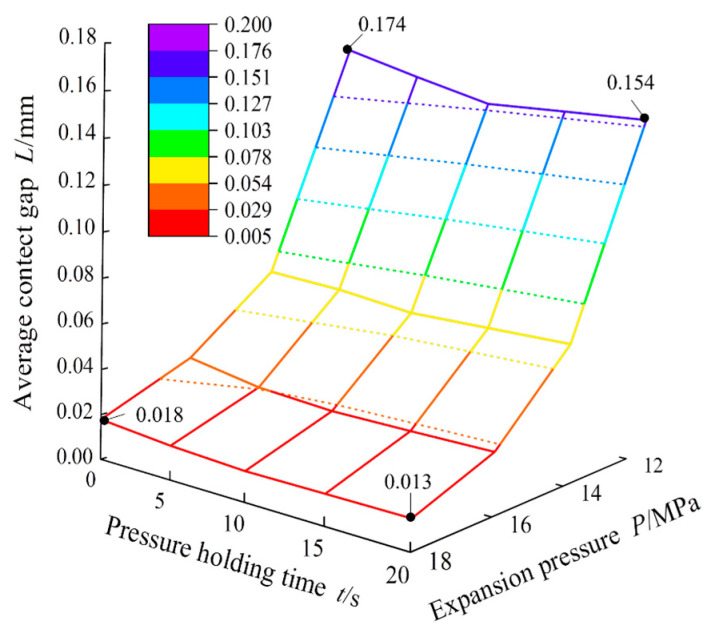
Influence of expansion pressure and holding time on average contact gap.

To analyse the variation law of the expansion pressure and pressure holding time for the average contact gap between the fin and the copper tube more intuitively, a three-dimensional surface coordinate diagram was drawn, as shown in Figure 11. Under the same pressure holding time, the average contact gap gradually decreased with increasing pressure. When the pressure holding time was 20 s and the expansion pressure increased from 12 to 18 MPa, the average contact gap distance decreased from 0.154 to 0.013 mm, and the average contact gap decreased by approximately 91.6%. Under the same expansion pressure, the average contact gap was largest when the pressure holding time was 0 s. After the pressure holding time increased to 10 s, the average contact gap remained basically unchanged, and the increase in the expansion pressure gradually reduced the influence of the pressure holding time on the contact gap. When the expansion pressure was 12 MPa and the holding time increased from 0 to 20 s, the average contact gap decreased from 0.174 to 0.154 mm, a decrease of 0.02 mm. When the expansion pressure was 18 MPa and the holding time increased from 0 to 20 s, the average contact gap decreased from 0.018 to 0.013 mm. The contact gap decreased by 0.005 mm, which is negligible. When the expansion pressure reached 18 MPa, there was no need to consider the holding time, and the minimum contact gap between the copper tube and the fin was obtained. When the expansion pressure increased from 12 to 18 MPa and the holding time increased from 0 to 20 s, the average contact gap distance decreased by 92.5%.

### 4.2. Variation in Contact Area

**Figure 12:**
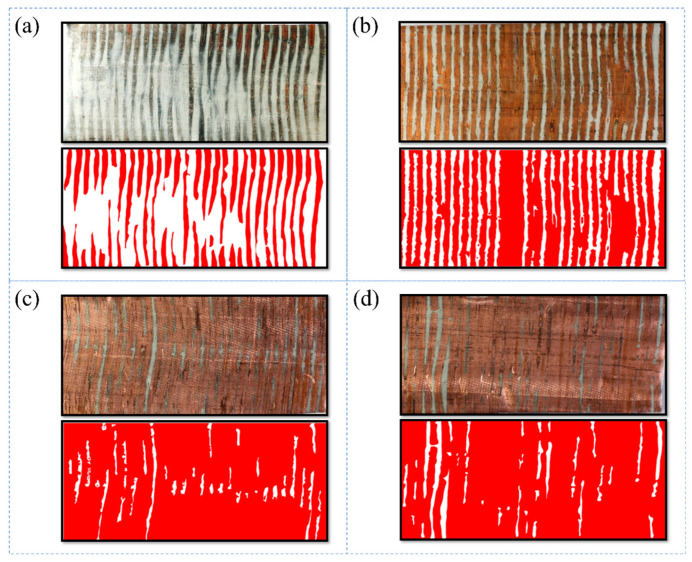
Spread distribution of contact area between copper tube and fin (expansion pressures of (**a**) 12, (**b**) 14, (**c**) 16, and (**d**) 18 MPa).

Figure 12 shows a distribution diagram of the contact area between the copper tube and the fin when the pressure holding time was 20 s. The total area of the copper tube was 1163 mm^2^. The red position is where the copper tube and fin were in contact, and the white position is where the copper tube and fin were not in contact. As can be seen from Figure 12a, when the expansion pressure was 12 MPa, the white uncontacted part was large, there was not complete contact between a single fin and the copper tube, and the contact area between the fin and the copper tube was relatively small. Compared with Figure 12b, it can be seen that when the expansion pressure was 14 MPa, a single fin could fully come into contact with the copper tube, but the fins were still not in contact with the copper tube, although the contact area increased. As can be seen from Figure 12c,d, when the expansion pressure was 16 MPa and 18 MPa, the fins were basically in contact with the copper pipe, and the contact area was large. The difference in contact area, between 16 MPa and 18 MPa, was found to be small. In order to obtain the contact area between the copper tube and the fin, the contact area and the contact area of the red position were calculated using ImageJ 1.53 software, as shown in Table 3. With an increase in the expansion pressure, the contact area and the proportion of the contact area increased continuously, and the increase rate of the contact area decreased continuously. When the expansion pressure reached 14 MPa, the contact area accounted for 56.1%. When the expansion pressure reached 18 MPa, the contact area accounted for 91.6%. The expansion pressure increased from 14 to 16 MPa, and the proportion of the contact area increased by approximately 34.9%. The expansion pressure increased from 16 to 18 MPa, and the proportion of the contact area only increased by approximately 0.6%.

**Figure 13:**
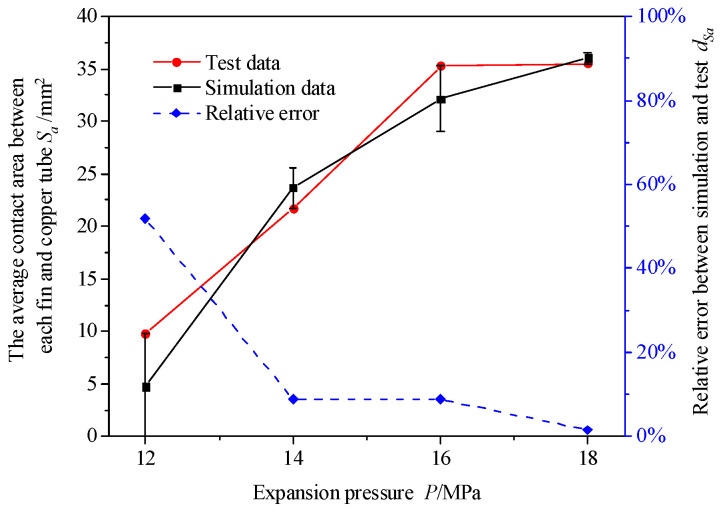
Comparison of the average contact areas between fin and copper tube in finite element simulation and experiment.

Because of the large error in the test control holding time, a finite element simulation was performed to study the variation rule of the holding time on the contact area more accurately. To reduce the calculation time, only five fins were installed on the copper tube for expansion in the finite element simulation. To compare the contact area of the test and verify the accuracy of the finite element model, the average contact area between each fin and copper tube was obtained by obtaining the average contact area of the test, as shown in Figure 13. The relative errors of the test data of the contact area and the finite element analysis results are shown in Table 4. *S* is the contact area between the copper tube and the fin, and *k* is the number of fins installed on the copper tube. The average contact area between each fin and the copper tube *S_a_* can be expressed as

(2) $$S_{a}=\frac{S}{k}$$

The variation law of the expansion pressure on the contact area obtained through finite element simulation was basically consistent with the test. The relative error between the test and finite element simulation results was less than 10%. However, the relative error in the contact area increased when the expansion pressure was 12 MPa. This was because when the contact area was measured using the chemical dip plating method, the contact area between the fin and copper tube side increased owing to its own gravity, resulting in a large relative error between the test and finite element simulation.

**Figure 14:**
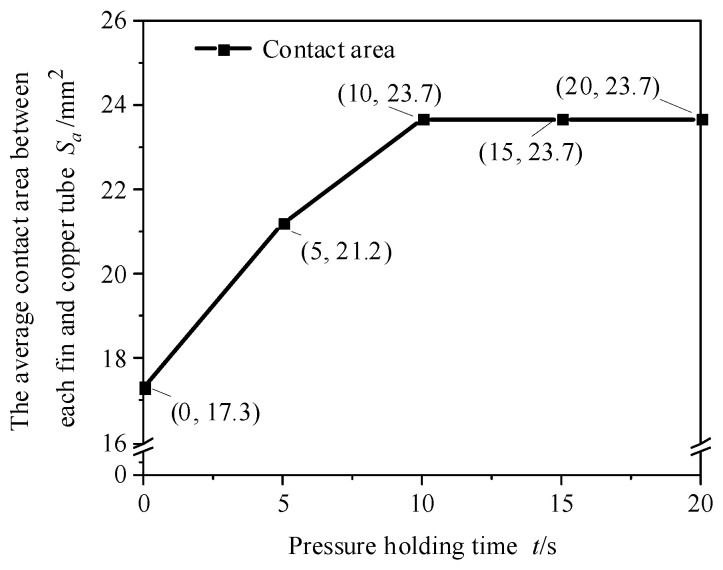
Average contact area between each fin and copper tube under different holding times (expansion pressure is 14 MPa).

The contact area between the copper tube and the fin with a typical expansion parameter combination (expansion pressure: 14 MPa, holding pressure time: 0–20 s) was analysed through finite element simulation. Figure 14 shows the average contact area between each fin and the copper tube under different holding times when the expansion pressure was 14 MPa. As shown in the figure, with an increase in pressure holding time, the contact area first increased and then remained unchanged. When the holding time exceeded 10 s, the holding time increased again, and the contact area remained unchanged. When the pressure holding time increased from 0 to 10 s, the average contact area between the fin and the copper tube increased from 17.3 to 23.7 mm^2^. After the pressure holding time reached 10 s and the pressure holding time increased, the contact area remained basically unchanged.

**Figure 15:**
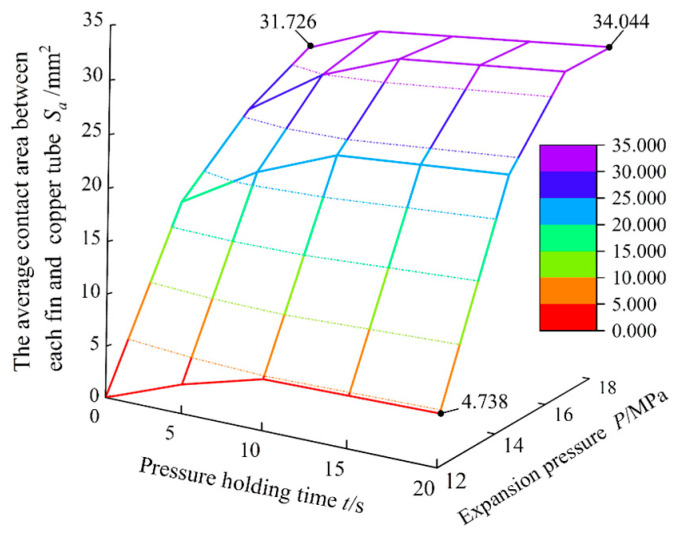
Influence of expansion pressure and holding time on the average contact area between each fin and copper tube.

For a more comprehensive and intuitive analysis of the variation in the average contact area between the fin and copper tube under the expansion pressure and pressure holding time, a three-dimensional surface coordinate diagram was drawn, as shown in Figure 15. With a constant pressure holding time, the average contact area between fin and copper tube increased with increasing expansion pressure, and the rate of increase gradually slowed. The calculation results show that when the expansion pressure increased from 12 to 14 MPa, the contact area increased by 399.6%, and the growth rate was faster. When the expansion pressure was increased from 14 to 16 MPa, the contact area increased by approximately 35.9%, and the growth rate decreased. From the overall perspective of the figure, when the expansion pressure was unchanged and the pressure holding time was 0–10 s, the contact area increased with increasing pressure holding time. However, with an increase in expansion pressure, the influence of the pressure holding time on the contact area gradually decreased. When the expansion pressure was 14 MPa, the contact area for a pressure holding time of 20 s was 36.7% greater than that for a pressure holding time of 0 s. When the expansion pressure was 18 MPa, the contact area for a pressure holding time of 20 s was only 7.3% higher than that for a pressure holding time of 0 s. Compared with the influence of the expansion pressure on the increase in contact area, the pressure holding time had little influence on the change in contact area. The expansion pressure was selected to be 18 MPa, and the maximum contact area was obtained when the pressure holding time exceeded 10 s.

## 5. Conclusions

In this study, hydraulic expansion technology was used to realise the assembly connection of a copper tube and fin. The influence of expansion pressure and holding time on contact gap and contact area was obtained through an experiment and finite element simulation. A method of chemical dipping was proposed to obtain the specific contact area value. The new contact area measurement method was used to obtain the direct measurement of the contact area between copper tube and fin and improved the reliability of the contact interface observation. The main conclusions of this study are as follows:

(1) The average contact gap decreased with increasing expansion pressure, and the rate of decrease tended to be gentle. An increase in the expansion pressure gradually reduced the influence of the holding pressure time on the contact gap. When the expansion pressure reached 18 MPa, there was no need to consider the pressure holding time, and the minimum contact gap between the copper tube and the fin was obtained.
(2) The contact area continued to increase with increasing expansion pressure, and the rate of increase continued to decrease. The contact area increased with an increase in the holding time. However, with an increase in the expansion pressure, the influence of the holding time on the contact area gradually decreased. The expansion pressure was 18 MPa and the maximum contact area was obtained when the pressure holding time was more than 10 s. When the expansion pressure was less than 18 MPa, the influence of the pressure holding time on the contact area must be considered.
(3) When the expansion pressure reached 18 MPa and the pressure holding time exceeded 10 s, the contact state between the copper tube and the fin both reached a minimum contact gap of 0.018 mm and the maximum contact area of 1105 mm^2^. Therefore, the best contact state between the copper tube and fin hydraulic expansion was reached with an expansion pressure of 18 MPa and a pressure holding time of more than 10 s.

However, the lack of hydraulic expansion forming equipment for integrated tube fin heat exchangers and the limitation of the measurement method of contact area technology may have led to certain limitations in the objectivity and universality of the research results. We should try our best to overcome this problem in future research on the hydraulic expansion forming of tube fin heat exchangers. In order to improve the accuracy and reliability of the test, the hydraulic expansion device and equipment of the tube fin radiator should be further improved.

## Figures and Tables

**Table 1:** Material characteristics of seamless internal threaded copper tube (TP2) and aluminium foil fins [22].

Materials	Elastic Modulus *E*/GPa	Poisson’s Ratio	Tensile Strength Rm/MPa	Yield Strength $\delta_{s}$/MPa
Aluminium foil fins	68	0.33	136.889	132
Seamless internal thread copper tube	127.36	0.33	205.807	66

**Table 2:** Relative error between test data and finite element analysis results for contact gap.

Expansion Pressure/Holding Time	12 MPa/20 s	14 MPa/20 s	16 MPa/20 s	18 MPa/20 s
The first group *L*_1_/mm	0.142	0.073	0.029	0.014
The second group *L*_2_/mm	0.121	0.082	0.032	0.018
The third group *L*_3_/mm	0.154	0.076	0.037	0.021
The average of the three groups *L_p_*/mm	0.139	0.077	0.031	0.018
Finite element simulation *L_F_*/mm	0.150	0.066	0.028	0.013
Relative error *δ*	7.9%	14.3%	9.7%	7.1%

**Table 3:** Contact area and proportion of contact area between copper tube and fin (total contact area: 1163 mm^2^).

Expansion Pressure *P*/MPa	12	14	16	18
Contact area *S*/mm^2^	295	653	1058	1065
Proportion of contact area *δ*_s_	25.4%	56.1%	91.0%	91.6%

**Table 4:** Relative error between test data and finite element analysis results for contact area.

Expansion Pressure/Holding Time	12 MPa/20 s	14 MPa/20 s	16 MPa/20 s	18 MPa/20 s
The first group *S*_1_/mm^2^	306	613	1143	1114
The second group *S*_2_/mm^2^	295	653	1058	1065
The third group *S*_3_/mm^2^	254	678	995	1137
The average of the three groups *S_p_*/mm^2^	285	648	1065	1105
Average of single fins *S_ap_*/mm^2^	9.5	21.6	35.5	36.8
Finite element simulation *S_F_*/mm^2^	4.7	23.7	32.2	36.0
Relative error *δ_Sa_*	50.5%	9.7%	9.3%	2.2%

## Data Availability

Data are contained within the article.
